# Acute Thoracic Aortic Dissection: A Retrospective Study at an Urban ED in Australia

**DOI:** 10.7759/cureus.114683

**Published:** 2026-08-17

**Authors:** Isabelle Ferarrio, Brian Burns

**Affiliations:** 1 Emergency Medicine, Northern Beaches Hospital, Sydney, AUS; 2 Faculty of Medicine and Health Sciences, Macquarie University, Sydney, AUS; 3 Faculty of Medicine and Health, The University of Sydney, Sydney, AUS

**Keywords:** ’ ‘acute aortic dissection, aortic dissection, chest pain, risk factors, thoracic aorta

## Abstract

Background

Thoracic aortic dissection (TAD) is a rare but often fatal condition if left undiagnosed. This study reviewed the detection rate and clinical presentations of TAD in an urban Australian ED.

Methods

A retrospective observational study was conducted from October 30, 2018 to March 30, 2023. The hospital imaging database (InteleViewer) was searched for radiology reports containing “aorta” or “aortic” together with “dissection” or the misspelling “disection.” The search identified 25 patients with acute TAD who presented to the ED. Medical records were reviewed, and the Aortic Dissection Detection Risk Score (ADD-RS) was applied retrospectively. Cases missed in the ED were also reviewed.

Results

Nineteen cases (76%) were type A, and six (24%) were type B, corresponding to 8.6 cases per 100,000 ED visits. Hypertension was the most common risk factor (64%). Pain presentations varied, with only five patients (20%) experiencing classic, sudden-onset central chest pain radiating to the back. TAD was diagnosed by CT aortography in 22 cases (88%). Chest radiography was performed in 12 patients and showed signs of TAD in six (50%). Bedside echocardiography was performed in 12 cases and detected TAD in one-third. The median time from ED arrival to surgical intervention was 230 minutes (IQR, 165-480 minutes). Fifteen patients (60%) had an ADD-RS ≤1; however, most did not undergo D-dimer testing. One case was initially missed in the ED but was subsequently diagnosed after hospital admission and had a favourable outcome.

Conclusions

Our study demonstrates the variability in the presenting features of TAD. Within this cohort, patients with confirmed TAD experienced short intervention times and favourable outcomes. The absence of classic symptoms and ADD-RS features does not exclude a diagnosis of TAD. Improving staff education regarding key symptoms and signs may enhance early detection.

## Introduction

Thoracic aortic dissection (TAD) is an uncommon but important diagnosis that is often missed and is associated with high morbidity and mortality, with reported mortality rates ranging from 12% to 34% [1-5]. These concerns have prompted the global patient-led Think Aorta campaign [6], which aims to increase awareness and improve the diagnosis of aortic dissection. With mortality reported to increase by 1-2% per hour following symptom onset in untreated patients [4,7], rapid detection and timely intervention are essential. Underdetection in emergency settings has been attributed to the rarity of the condition, the lack of a validated clinical decision tool, and insufficient education among medical staff [2]. Additionally, its presentation overlaps with that of other conditions, such as acute coronary syndromes, meaning that misdiagnosis can lead to worse outcomes [8].

Large multicentre studies and databases have provided important insights into the presentation and management of TAD, highlighting areas for improvement in research and clinical diagnosis. The Aortic Dissection Detection Risk Score (ADD-RS) [9] has also been developed and analysed [5,10]. It incorporates 12 high-risk markers across three categories and generates a score ranging from 0 to 3. When combined with D-dimer testing, it can help guide decisions regarding dedicated CT imaging of the aorta. Recent work has further highlighted areas for improvement in research and clinical diagnosis while also questioning the validity of the ADD-RS [2]. Overall, these efforts have led to increased awareness of TAD and its presentation, but little has been published about this condition in Australian emergency settings.

The primary aim of this study was to review the detection rate and presenting characteristics of TAD in an urban Australian ED. The secondary aims were to analyse the time to intervention in our cohort and explore the applicability of the ADD-RS to our patient population.

## Materials and methods

Study population

**Figure 1:**
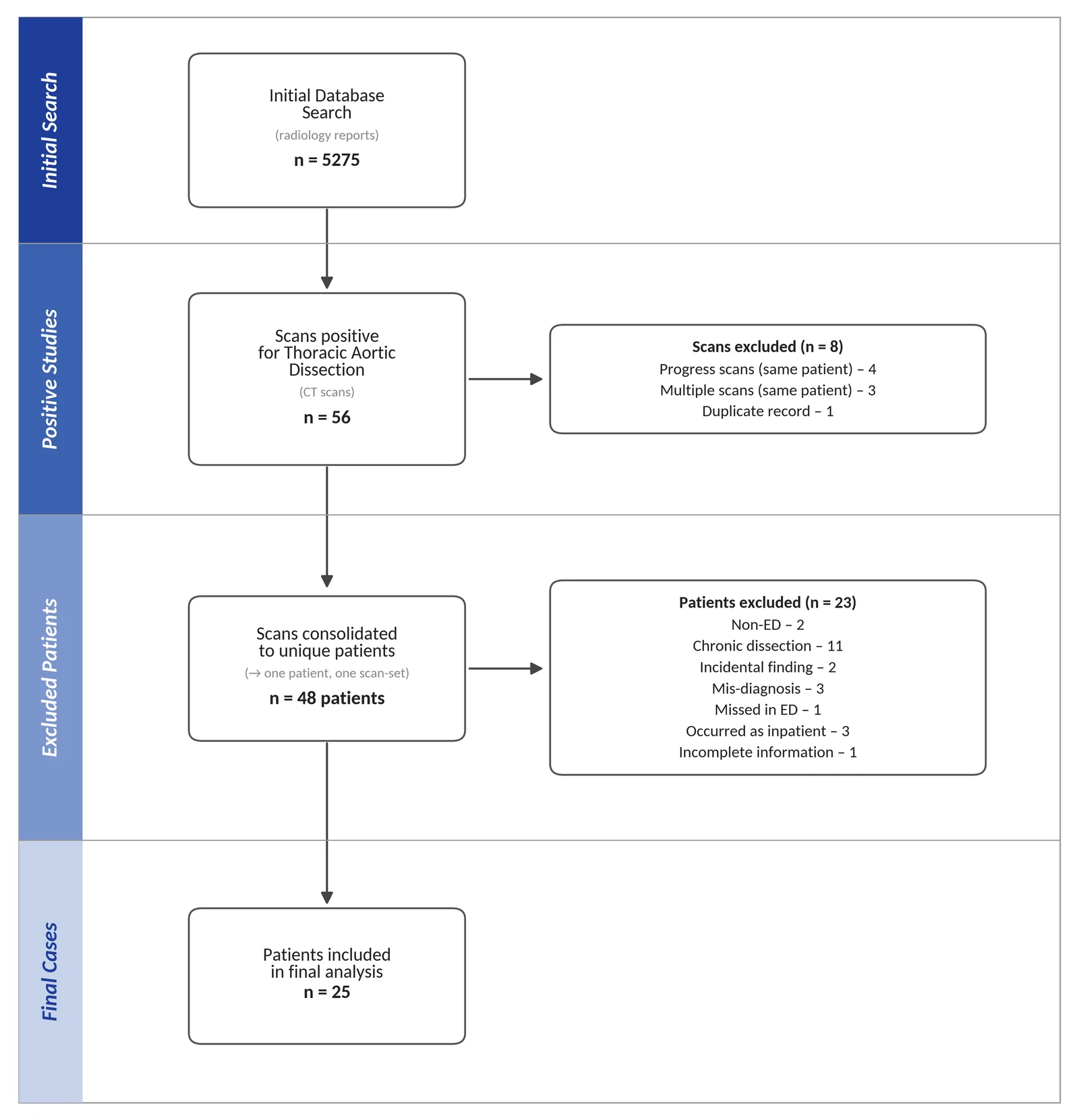
Study selection flowchart showing the inclusion and exclusion of thoracic aortic dissection (TAD) cases presenting to the ED.

This was a retrospective observational study conducted at the Northern Beaches Hospital ED, which has an annual census of approximately 70,000 attendances. To identify potential cases, the ED imaging database (InteleViewer) was searched for all radiology reports generated between October 30, 2018 and March 30, 2023 that contained the terms “aorta” or “aortic” in combination with “dissection” or “disection,” with the latter spelling included to capture typographical errors in the reports. This initial search returned 5,275 results, which were then filtered to include only CT reports. The screening was undertaken by a single assessor (IF), with a second assessor (BB) consulted when a report was unclear. This process identified 56 positive CT scans, which were subsequently reduced to 25 eligible cases of acute Stanford type A or type B TAD presenting to the ED (Figure 1).

Cases were eligible for inclusion if they involved acute TAD (type A or type B) presenting to the ED within the study timeframe. Cases were excluded for the following reasons: duplicate records; multiple scans of the same patient, including progress scans; chronic TAD; cases not identified in the ED; events occurring during an inpatient admission; misdiagnoses; incidental findings; or insufficient information for inclusion in the study.

Chart review was performed in accordance with the methodology described by Gilbert EH et al. [11]. In accordance with this methodology, cases were identified using prespecified inclusion and exclusion criteria, as described above, and data were abstracted onto a standardised case report form with explicit variable definitions (see Data variables and Data definitions). As data abstraction was performed by a single reviewer, criteria relating to abstractor training, monitoring, and interrater reliability were considered not applicable to this study; this is noted as a limitation. Results were collated in Microsoft Excel (Microsoft Corporation, Redmond, WA, USA) for analysis. Reporting was conducted in accordance with the STROBE checklist [12].

Data variables

Medical records were reviewed by a single abstractor (IF). The variables collected included patient demographics (age and sex), medical history, mode and timing of presentation, presenting symptoms, clinical signs, laboratory and radiological findings, interventions undertaken, and time to intervention.

Data definitions

Several time intervals were defined for the purposes of this study. Time to presentation was defined as the interval from patient-reported symptom onset to arrival at ED triage, and time to CT aortography was defined as the interval from arrival at ED triage to CT acquisition. Time to intervention was considered in two ways for patients managed surgically: as the interval from arrival at ED triage to intervention and as the interval from patient-reported symptom onset to intervention.

Statistics

Data were entered, managed, and collated in Microsoft Excel (Microsoft Corporation, Redmond, WA, USA), which was also used for all statistical analyses. Given the descriptive nature of the study and the limited sample size, the analysis was purely descriptive, and no formal comparative or inferential statistical testing was undertaken. Continuous and ordinal data were summarised using the median and IQR, and categorical data were expressed as frequencies and percentages. No imputation was performed for missing data; where information was unavailable, this was reflected in the relevant denominators.

Application of the ADD-RS

Chart review was performed to apply the ADD-RS [9], which generates a score from 0 to 3 based on the presence of markers in three categories: high-risk conditions, high-risk pain features, and high-risk examination features, with one point assigned per category. D-dimer results, where available, were reviewed to combine the score with the algorithm proposed by Nazerian P et al., which uses D-dimer testing in patients with an ADD-RS ≤1 to guide decisions regarding imaging [10].

Ethics

The project was approved by the NBH LNR/QI Project Review Subcommittee and was deemed to involve low or negligible risk. The approval number was 2023/NBH/015.

## Results

Patient characteristics

Patient characteristics are presented in Table 1. Nineteen patients (76%) presented with type A dissection and six (24%) with type B dissection. Hypertension was the most common risk factor, affecting 16 patients (64%), followed by hypercholesterolaemia in nine (36%) and previous or current smoking in six (24%). Pain presentations were variable; only five patients (20%) presented with classic sudden-onset central chest pain radiating to the back. The median systolic blood pressure (SBP) was 131 mmHg (IQR, 99-170 mmHg). Only five patients (20%) had an upper-limb BP differential.

**Table 1:** Patient characteristics. † HTN: Hypertension; ‡ AAA: Abdominal aortic aneurysm; § CVA: Cerebrovascular accident; ¶ SBP: Systolic blood pressure.

Patient characteristics	Total (n = 25)	Type A (n = 19)	Type B (n = 6)
Age, years, median (IQR)	73 (62-78)	71 (59-78)	74.5 (62-81)
Male	17 (68%)	13 (68.4%)	4 (66.7%)
Female	8 (32%)	6 (31.6%)	2 (33.3%)
Mode of presentation			
Walk-in	6 (24%)	4 (21.1%)	2 (33.3%)
Ambulance	19 (76%)	15 (78.9%)	4 (66.7%)
Risk factors			
HTN†	16 (64%)	13 (68.4%)	3 (50%)
Hypercholesterolaemia	9 (36%)	6 (31.6%)	3 (50%)
Previous or current smoker	6 (24%)	5 (26.3%)	1 (16.7%)
Previous dissection	4 (16%)	4 (21.1%)	0 (0%)
Ischaemic heart disease	4 (16%)	2 (10.5%)	2 (33.3%)
Known AAA‡	3 (12%)	3 (15.8%)	0 (0%)
Previous cardiovascular surgery	1 (4%)	1 (5.3%)	0 (0%)
Obesity	1 (4%)	1 (5.3%)	0 (0%)
CVA§	1 (4%)	0 (0%)	1 (16.7%)
Diabetes mellitus	1 (4%)	1 (5.3%)	0 (0%)
Systolic blood pressure			
Median (IQR), mmHg	131 (99-170)	110 (95-147)	229 (156-240)
Hypertensive (SBP¶ >140 mmHg)	10 (40%)	6 (31.6%)	4 (66.7%)
Normotensive	10 (40%)	8 (42.1%)	2 (33.3%)
Hypotensive (SBP¶ <90 mmHg)	5 (20%)	5 (26.3%)	0 (0%)
Clinical signs			
Upper-limb blood pressure difference (>20 mmHg)	5 (20%)	3 (15.8%)	2 (33.3%)
Pulse deficit	2 (8%)	2 (10.5%)	0 (0%)
Hypoxia	2 (8%)	2 (10.5%)	0 (0%)
Tachypnoea	2 (8%)	1 (5.3%)	1 (16.7%)
Neurological deficit	2 (8%)	2 (10.5%)	0 (0%)
Limb ischaemia	1 (4%)	1 (5.3%)	0 (0%)
Systolic murmur	3 (12%)	1 (5.3%)	2 (33.3%)
Muffled heart sounds	1 (4%)	1 (5.3%)	0 (0%)
Pain location			
Chest pain	17 (68%)	14 (73.7%)	3 (50%)
Central	5 (20%)	3 (15.8%)	2 (33.3%)
Left-sided	1 (4%)	1 (5.3%)	0 (0%)
Right-sided	1 (4%)	1 (5.3%)	0 (0%)
Upper back pain	2 (8%)	1 (5.3%)	1 (16.7%)
Lower back pain	2 (8%)	1 (5.3%)	1 (16.7%)
Abdominal pain	5 (20%)	2 (10.5%)	3 (50%)
Jaw pain	4 (16%)	4 (21.1%)	0 (0%)
Pain descriptors			
Sharp	2 (8%)	1 (5.3%)	1 (16.7%)
Dull	1 (4%)	1 (5.3%)	0 (0%)
Heavy	2 (8%)	2 (10.5%)	0 (0%)
Tight	1 (4%)	1 (5.3%)	0 (0%)
Pain timing			
Sudden-onset pain	11 (44%)	9 (47.4%)	2 (33.3%)
Radiation of pain			
Radiation to the back	7 (28%)	5 (26.3%)	2 (33.3%)
Radiation to the legs	2 (8%)	1 (5.3%)	1 (16.7%)
Radiation to the abdomen	0 (0%)	0 (0%)	0 (0%)
Radiation to the left arm	1 (4%)	1 (5.3%)	0 (0%)
Radiation to the neck	1 (4%)	1 (5.3%)	0 (0%)
Classical presentation			
Sudden-onset central chest pain radiating to the back	5 (20%)	3 (15.8%)	2 (33.3%)

Diagnostic test findings

Diagnostic test and imaging results are presented in Table 2. ECG was performed in 23 patients (92%), of whom seven (30.4%) demonstrated ischaemic changes. Chest radiography (CXR) was performed in 12 patients (48%), six (50%) of whom had a widened mediastinum. Bedside clinician-performed echocardiography (ECHO) was performed in 12 patients (48%), and four of these patients (33.3%) demonstrated findings consistent with TAD. D-dimer testing was performed in four patients (16%) and produced an elevated result in three. Troponin testing was performed in 20 patients (80%), of whom three (15%) had elevated troponin levels.

**Table 2:** Diagnostic tests and imaging results. CXR: Chest X-ray; ECHO: Echocardiography.

Diagnostic test and result	n (%)
ECG	
Performed	23 (92%)
Normal ECG	16 (69.6%)
Ischaemia on ECG	7 (30.4%)
Not documented	2 (8%)
CXR	
Performed	12 (48%)
Widened mediastinum	6 (50%)
Normal	6 (50%)
Not performed	13 (52%)
Bedside ECHO	
Performed	12 (48%)
Findings consistent with dissection	4 (33.3%)
Enlarged aortic root	2 (16.7%)
Dissection flap	1 (8.3%)
Cardiac tamponade	1 (8.3%)
No findings of dissection	8 (66.7%)
Not performed	13 (52%)
D-dimer	
Performed	4 (16%)
Raised (≥0.50 μg/mL FEU)	3 (75%)
Negative (<0.50 μg/mL FEU)	1 (25%)
Not performed	21 (84%)

Clinical course, management, and outcomes

The timing of intervention, clinical course, and outcomes are presented in Tables 3-4. Of the 19 patients with type A TAD, 15 (78.9%) underwent urgent surgical intervention, and one patient died following surgery. Of the six patients with type B TAD, one underwent surgical intervention, while the remaining five were managed medically. One patient died following medical management.

**Table 3:** Clinical course and outcomes.

Intervention type and outcome	Total (n = 25)	Type A (n = 19)	Type B (n = 6)
Medical management	5 (20%)	0 (0%)	5 (83.3%)
Mortality following medical management	1 (20%)	-	1 (20%)
Surgical intervention	16 (64%)	15 (78.9%)	1 (16.7%)
Mortality following surgical intervention	1 (6.3%)	1 (6.7%)	0 (0%)
Palliative management	4 (16%)	4 (21.1%)	0 (0%)
Overall mortality	6 (24%)	5 (26.3%)	1 (16.7%)
Length of stay, median (IQR), days	10 (7.25-13.5)	10 (8.4-16)	10 (10-10)

**Table 4:** Timing of diagnostic testing and intervention.

Time interval	Median (IQR), minutes
Time to presentation	75 (60-112.5)
Time to CT aortogram	90 (60-191.25)
Time to intervention	
ED presentation to intervention	230 (165-480)
Symptom onset to intervention	330 (221.25-632.5)

ADD-RS score

We retrospectively applied the ADD-RS to our study population (Table 5) [9]. Among patients with an ADD-RS ≤1, for whom the proposed algorithm recommends D-dimer testing before proceeding to imaging, one patient had a negative D-dimer result. However, this patient had a positive bedside ECHO that visualised a dissection flap and an enlarged aortic root, suggesting TAD. D-dimer testing was not performed in the majority of patients; therefore, the combined ADD-RS/D-dimer algorithm proposed by Nazerian et al. could not be fully applied [10].

**Table 5:** ADD-RS scores. † D-dimer level ≥0.50 μg/mL FEU; ‡ D-dimer level <0.50 μg/mL FEU. ADD-RS: Aortic Dissection Detection Risk Score.

ADD-RS score	Total (n = 25)	Type A (n = 19)	Type B (n = 6)
Score 0	2 (8%)	1 (5.3%)	1 (16.7%)
Score 1	13 (52%)	9 (47%)	4 (66.6%)
Score 2	6 (24%)	5 (26.3%)	1 (16.7%)
Score 3	4 (16%)	4 (21%)	0 (0%)
D-dimer results among patients with ADD-RS ≤1			
ADD-RS ≤1	15 (60%)	10 (52.6%)	5 (83.3%)
D-dimer not performed	12 (80%)	7 (70%)	5 (100%)
D-dimer elevated†	2 (13.3%)	2 (20%)	0 (0%)
D-dimer negative‡	1 (6.7%)	1 (10%)	0 (0%)

## Discussion

Prevalence and Mortality

There were 25 cases of acute TAD during the 4.5-year study period. This corresponds to approximately 8.6 cases per 100,000 presentations to our ED. This is comparable to Australian data reporting rates of TAD ranging from 3.31 to 9.5 per 100,000 ED presentations [2, 13].

The overall in-hospital mortality rate was 24%, with four patients receiving palliative care in the ED because of their age and comorbidities. This is similar to data estimating the mortality rate of TAD to be between 12% and 34% [1-5, 14]. One patient died following surgical intervention. This patient was transitioned to palliative care during the postoperative period after suffering a large middle cerebral artery (MCA) stroke that was considered unsurvivable. One patient died following medical management. This patient had a Type B dissection that progressed to Type A following medical management and was transitioned to palliative care after multiple large-territory infarcts were identified during the postoperative period.

With mortality reported to increase by 1-2% per hour following symptom onset in untreated patients [4, 7], rapid detection and timely intervention are essential. The median time from presentation to the ED to surgical intervention was 230 minutes (IQR, 165-480). Although our hospital is a tertiary centre, it does not have an emergency cardiothoracic surgery service; therefore, patients presenting to our ED require transfer to a nearby tertiary cardiothoracic centre. Despite this, the time to intervention was short, and the mortality rate following surgical intervention was 6.24% (1/16 patients). The mortality rate for Type A TAD was low compared with the rates reported in larger studies, which range from 9.1% to 19.5% [5, 15, 16]. This could be attributed to several factors, including a shorter time to diagnosis and intervention, differences in case mix, or chance given the small study population.

Pain characteristics

Pain rarely presented as the classically taught “tearing chest pain radiating through to the back,” with only 20% of our patients describing these symptoms at presentation. The literature suggests that “ripping” or “tearing” are also uncommon descriptors and are not strong predictors of TAD [5, 9, 17]; neither was reported in our study population.

Although chest pain is the most common symptom (79%) [5], it has not been found to be a strong predictor of TAD (positive likelihood ratio (LR+), 1.02-1.12) [17]. Chest pain was the most commonly reported symptom in our study population (68%), followed by abdominal pain (20%) and jaw pain (16%). Data from the International Registry of Acute Aortic Dissection (IRAD) suggest that chest pain is more commonly associated with Type A dissection and back pain with Type B dissection [5]. In our study population, chest pain was reported more frequently by patients with Type A dissections than by those with Type B dissections (73.7% vs. 50%); however, back pain was documented in both groups. Our data suggest that abdominal pain was more commonly associated with Type B dissections (50% vs. 10.5%), whereas jaw pain was more commonly associated with Type A dissections (21.1% vs. 0%). These patterns may reflect the anatomical differences between Type A and Type B dissections.

Dissections can also present in the absence of pain (6-11%) [5, 18], with one patient (4%) in our study reporting no pain. However, this patient had severe dementia, making symptom reporting unreliable.

Signs of aortic dissection

The signs with the highest reported specificity for TAD include hypotension (95%), focal neurological deficit (95%), and pulse deficit (91%) [17]. In our study population, the median SBP was 131 mmHg (IQR, 99-170), with five patients (20%) presenting with hypotension (SBP <90 mmHg) and, more commonly, 10 patients (40%) presenting with hypertension (SBP >140 mmHg). Focal neurological deficit was present in two patients (8%), and pulse deficit was reported in two patients (20%). Although these signs have reported specificities of >90%, they are not sufficient on their own to establish a diagnosis of TAD.

Rogers AM et al. examined data from IRAD and found that the most common signs of TAD were a new murmur of aortic insufficiency in conjunction with pain (23.6%) and a pulse deficit or BP differential [9]. BP differentials (SBP difference >20 mmHg) were documented in 20% of our study population. Bilateral BP measurements were not recorded for all patients; therefore, the frequency of this finding may have been underestimated.

Because of the diversity of the pathology included in TAD, symptoms and signs can vary depending on the region of the vessel affected. As a result, it is important to consider TAD as a cause of a patient’s symptoms, even in the absence of the classically described presentation. It is important to look for common signs of TAD, including pulse deficit, neurological deficit, BP differential, and a new murmur of aortic insufficiency, in any patient in whom TAD is being considered [2]; however, the absence of these findings does not reliably exclude a diagnosis of TAD. Increasing the use of clinician-performed bedside USG as an extension of the clinical examination may improve the detection of TAD.

Detection rates

Other conditions that may present similarly to TAD include acute coronary syndrome (ACS) and pulmonary embolism (PE), both of which may involve thrombolytic or anticoagulant therapy as part of their treatment. The administration of antithrombotic agents to patients with TAD could be potentially devastating [8], increasing the importance of prompt and accurate detection in the emergency setting.

A systematic review of 1,663 patients found a misdiagnosis rate of 33.8% [19]. Factors contributing to misdiagnosis reported in the literature include walk-in mode of admission, features suggestive of another disease, a lack of typical features such as a widened mediastinum on chest X-ray, and anterior chest pain [8, 18, 19]. A systematic review also found that factors associated with an accurate diagnosis included comprehensive history-taking and increased use of imaging [19].

During our search, we identified one case of misdiagnosis. This patient presented with chest pain and serially elevated troponin levels and was treated for a non-ST-elevation myocardial infarction (NSTEMI). After subsequent episodes of chest pain, the patient was referred for a ventilation/perfusion (V/Q) scan. A computed tomography pulmonary angiogram (CTPA) performed in the ED had been reported as negative; however, the reporting radiologist re-reviewed the initial CTPA images and identified dilatation of the aorta. The diagnosis was subsequently confirmed by CT aortogram. With nearly one in three cases of TAD being misdiagnosed, this further highlights the importance of education regarding the presentation and impact of this condition [8, 19].

Risk factors

Hypertension is the most commonly associated risk factor for TAD, with its prevalence reported to range from 45% to 100% [5, 20]. People with hypertension are four times more likely to develop TAD than the normotensive population [3, 4, 20], and a prospective study found that the population-attributable risk of TAD due to hypertension was 54% [3]. The prevalence of hypertension in our study population was 64%, in keeping with the literature.

Another common risk factor among the study population was current or previous smoking (24%). Smoking is frequently reported as a risk factor for TAD, with the prevalence of “ever smokers” reported to be 61.5% in the prospective Oxford Vascular Study [21]. Another large prospective study found that the proportion of TAD risk attributable to smoking was 14% [3].

The ADD-RS lists known thoracic aortic aneurysm (TAA) as a high-risk condition, and its prevalence in the IRAD data was 14.7%. In our study, no patients had a known TAA; however, three patients (12%) had a known abdominal aortic aneurysm (AAA). IRAD data also suggest that marked pre-existing aneurysmal dilatation is not always present before a dissection event. IRAD data showed that 60% of patients had a maximum aortic diameter of <5.5 cm, the current threshold for elective surgical repair, and 40% had an aortic diameter of <5.0 cm [5]. Larger aortic diameters were found to coexist with conditions such as Marfan syndrome, bicuspid aortic valve, and previous coronary artery bypass surgery, suggesting that the aortic diameter threshold for elective repair should be lowered for patients without these conditions.

Considering the presence of these risk factors is essential when TAD is suspected and, together with other presenting signs and symptoms, may help determine which patients require further investigation.

Laboratory tests

D-dimer has been proposed as a biomarker for the detection of TAD and is now included in the ADD-RS pathway. The sensitivity of D-dimer for TAD, using a cutoff value of 0.50 μg/mL FEU, has been reported to range from 94% to 98%; however, its specificity is low, ranging from 41% to 64% [10, 22-24]. Nazerian P et al. suggested that a negative D-dimer result combined with an ADD-RS of 0 could be sufficient to rule out TAD. This approach had a failure rate of 0.3% (95% CI, 0.1-1.0%) in their study of 1,850 patients [10].

D-dimer was not frequently measured in our study population, with only four of 25 patients (16%) undergoing D-dimer testing. No patients met the rule-out criterion of an ADD-RS of 0 and a negative D-dimer result, and among those who had an ADD-RS of 0, one had a raised D-dimer level. These findings should be interpreted with caution because D-dimer levels were measured in very few patients and only confirmed TAD cases were included. Further studies evaluating D-dimer levels in patients without TAD are needed to better assess its utility as a rule-out test.

Imaging

CT aortogram is the preferred diagnostic method for TAD because of its high sensitivity and specificity, ranging from 95% to 100% [25, 26], and its widespread availability, even in rural areas. Its use must be balanced against potential risks, including radiation exposure, allergic reactions, and renal injury.

In total, 1,166 CT scans specifically evaluating the thoracic aorta were performed in the ED during the study period. Twenty-two of the 25 patients were identified through these scans, with the other three patients identified using CT of the abdomen and pelvis, CTPA, and bedside echocardiography. This represents a positive imaging yield of 1.89%, which is below the suggested imaging yield of 3% reported by Kelly AM et al. [2]. Liberal CT scanning in EDs is increasing in high-income countries. A cost-effectiveness analysis of CT scanning in our cohort was beyond the scope of this retrospective review.

ECHO

Point-of-care echocardiography is increasingly being used by clinicians in the ED, particularly for the assessment of shock. However, it should be used with caution when attempting to exclude aortic dissection. We recommend that when aortic dissection is suspected, a formal CT aortogram should be performed in addition to bedside echocardiography.

ADD-RS

The ADD-RS [9, 27] was developed to determine a patient’s risk of aortic dissection at the bedside based on 12 clinical risk markers, including presenting features, pre-existing conditions, and physical examination findings. Although the ADvISED trial found a low failure rate of 0.3% when the ADD-RS was combined with a negative D-dimer result (<0.5 μg/mL FEU) [10], concerns remain regarding its validation and testing in its intended population. Kelly AM et al. found that using the ADD-RS would substantially increase the number of CT scans performed [2].

The limited use of D-dimer measurement in our cohort made it difficult to assess the performance of this score in our patient population. Although the ADD-RS has not been robustly tested, the features it highlights may assist physicians in deciding whether to investigate for TAD. Recent recommendations in the UK suggest that, in the absence of an alternative diagnosis, patients presenting with high-risk features outlined in the ADD-RS should be considered for CT aortography [28].

Limitations

Our study was limited by its retrospective design and small sample size. In addition, selection bias was a limitation because only confirmed cases of TAD were included, making the findings inapplicable to the broader population of patients presenting to the ED with undifferentiated chest pain. Our case-identification strategy relied solely on a search of radiology reports. This approach assumes that every acute TAD case underwent CT imaging and had a report containing our search terms. Cases diagnosed using alternative modalities, transferred before imaging, or not undergoing CT may therefore have been missed, and the completeness of case ascertainment cannot be guaranteed. As data abstraction was performed by a single abstractor, with a second reviewer consulted only in cases of uncertainty, there is a risk of abstraction bias that we were unable to quantify. The limited use of a standardised pathway meant that documentation and diagnostic testing were not uniform across all patients. This likely reflects real-world emergency medicine practice. It is also difficult to extrapolate these findings to resource-limited settings, where access to CT imaging may be financially constrained.

## Conclusions

In conclusion, our study demonstrates the frequency of TAD and the variability of its presenting symptoms and signs, with relatively few patients presenting with the classically described features. Within this cohort, patients with confirmed TAD experienced short times to intervention and favourable outcomes. The ADD-RS highlights key features of TAD of which all physicians should be aware; however, the absence of these features should not be used to exclude TAD. We identified areas for improvement in staff education, including increasing awareness of the condition and its key symptoms and signs.

## References

[1] Diercks DB, Promes SB, Schuur JD, Shah K, Valente JH, Cantrill SV (2015). Clinical policy: critical issues in the evaluation and management of adult patients with suspected acute nontraumatic thoracic aortic dissection. Ann Emerg Med.

[2] Kelly AM, Lee XQ, Curran J (2023). What can be done to improve diagnosis of aortic dissection?. Emerg Med Australas.

[3] Landenhed M, Engström G, Gottsäter A (2015). Risk profiles for aortic dissection and ruptured or surgically treated aneurysms: a prospective cohort study. J Am Heart Assoc.

[4] Gawinecka J, Schönrath F, von Eckardstein A (2017). Acute aortic dissection: pathogenesis, risk factors and diagnosis. Swiss Med Wkly.

[5] Evangelista A, Isselbacher EM, Bossone E (2018). Insights from the international registry of acute aortic dissection: a 20-year experience of collaborative clinical research. Circulation.

[6] (2024). Think Aorta. https://www.thinkaorta.net/.

[7] Erbel R, Alfonso F, Boileau C (2001). Diagnosis and management of aortic dissection. Eur Heart J.

[8] Hansen MS, Nogareda GJ, Hutchison SJ (2007). Frequency of and inappropriate treatment of misdiagnosis of acute aortic dissection. Am J Cardiol.

[9] Rogers AM, Hermann LK, Booher AM (2011). Sensitivity of the aortic dissection detection risk score, a novel guideline-based tool for identification of acute aortic dissection at initial presentation: results from the International Registry of Acute Aortic Dissection. Circulation.

[10] Nazerian P, Mueller C, Soeiro AM (2018). Diagnostic accuracy of the aortic dissection detection risk score plus D-dimer for acute aortic syndromes: the ADvISED Prospective Multicenter Study. Circulation.

[11] Gilbert EH, Lowenstein SR, Koziol-McLain J, Barta DC, Steiner J (1996). Chart reviews in emergency medicine research: Where are the methods?. Ann Emerg Med.

[12] von Elm E, Altman DG, Egger M, Pocock SJ, Gøtzsche PC, Vandenbroucke JP (2008). The Strengthening the Reporting of Observational Studies in Epidemiology (STROBE) statement: guidelines for reporting observational studies. J Clin Epidemiol.

[13] Dinh MM, Bein KJ, Delaney J, Berendsen Russell S, Royle T (2020). Incidence and outcomes of aortic dissection for emergency departments in New South Wales, Australia 2017-2018: a data linkage study. Emerg Med Australas.

[14] Evangelista A, Rabasa JM, Mosquera VX (2018). Diagnosis, management and mortality in acute aortic syndrome: results of the Spanish Registry of Acute Aortic Syndrome (RESA-II). Eur Heart J Acute Cardiovasc Care.

[15] Reutersberg B, Salvermoser M, Trenner M, Geisbüsch S, Zimmermann A, Eckstein HH, Kuehnl A (2019). Hospital incidence and in‐hospital mortality of surgically and Interventionally treated aortic dissections: secondary data analysis of the nationwide German diagnosis‐related group statistics from 2006 to 2014. J Am Heart Assoc.

[16] Higgins J, Lee MK, Co C, Janusz MT (2014). Long-term outcomes after thoracic aortic surgery: a population-based study. J Thorac Cardiovasc Surg.

[17] Ohle R, Kareemi HK, Wells G, Perry JJ (2018). Clinical examination for acute aortic dissection: a systematic review and meta‐analysis. Acad Emerg Med.

[18] Kurabayashi M, Miwa N, Ueshima D (2011). Factors leading to failure to diagnose acute aortic dissection in the emergency room. J Cardiol.

[19] Lovatt S, Wong CW, Schwarz K (2022). Misdiagnosis of aortic dissection: a systematic review of the literature. Am J Emerg Med.

[20] Zhou Z, Cecchi AC, Prakash SK, Milewicz DM (2022). Risk factors for thoracic aortic dissection. Genes (Basel).

[21] Howard DP, Banerjee A, Fairhead JF, Perkins J, Silver LE, Rothwell PM (2013). Population-based study of incidence and outcome of acute aortic dissection and premorbid risk factor control: 10-year results from the Oxford Vascular Study. Circulation.

[22] Watanabe H, Horita N, Shibata Y, Minegishi S, Ota E, Kaneko T (2016). Diagnostic test accuracy of D-dimer for acute aortic syndrome: systematic review and meta-analysis of 22 studies with 5000 subjects. Sci Rep.

[23] Asha SE, Miers JW (2015). A systematic review and meta-analysis of D-dimer as a rule-out test for suspected acute aortic dissection. Ann Emerg Med.

[24] Yao J, Bai T, Yang B, Sun L (2021). The diagnostic value of D-dimer in acute aortic dissection: a meta-analysis. J Cardiothorac Surg.

[25] Baliga RR, Nienaber CA, Bossone E (2014). The role of imaging in aortic dissection and related syndromes. JACC Cardiovasc Imaging.

[26] Shiga T, Wajima Z, Apfel CC, Inoue T, Ohe Y (2006). Diagnostic accuracy of transesophageal echocardiography, helical computed tomography, and magnetic resonance imaging for suspected thoracic aortic dissection: systematic review and meta-analysis. Arch Intern Med.

[27] Hiratzka LF, Bakris GL, Beckman JA (2010). 2010 ACCF/AHA/AATS/ACR/ASA/SCA/SCAI/SIR/STS/SVM guidelines for the diagnosis and management of patients with Thoracic Aortic Disease: a report of the American College of Cardiology Foundation/American Heart Association Task Force on Practice Guidelines, American Association for Thoracic Surgery, American College of Radiology, American Stroke Association, Society of Cardiovascular Anesthesiologists, Society for Cardiovascular Angiography and Interventions, Society of Interventional Radiology, Society of Thoracic Surgeons, and Society for Vascular Medicine. Circulation.

[28] Callaway M, Redfern E, France J (2024). Diagnosis of Thoracic Aortic Dissection in the Emergency Department. Royal College of Radiologists.

